# Title: A Qualitative Systematic Review of Parental Perceptions, Motivators, and Barriers to Management of Childhood Obesity

**DOI:** 10.2147/DMSO.S490475

**Published:** 2024-12-11

**Authors:** Sarah Musa, Ayman Al-Dahshan, Vahe Kehyayan

**Affiliations:** 1 Department of Preventative Health, Primary Health Care Corporation, Doha, Qatar; 2 Preventive Medicine Division, Department of Medicine, Hamad Medical Corporation, Doha, Qatar; 3 Department of Healthcare Management, College of Business, University of Doha for Science & Technology, Doha, Qatar

**Keywords:** obesity, children, parents, barriers

## Abstract

**Background:**

Childhood obesity is a global epidemic affecting millions worldwide. Children living with obesity face increased risks of health-related and psychosocial problems extending into adulthood. Parents and carers play a crucial role in cultivating healthy habits in their children. This review aims to synthesize qualitative research on parental perceptions, motivators, and barriers in managing childhood obesity and their views on weight management programs.

**Methods:**

This systematic review was performed in accordance with the guidelines established by the Preferred Reporting Items for Systematic Reviews and Meta-Analyses. A variety of electronic databases were explored for qualitative studies published between 2006 and 2023. The CASP checklist was employed to assess the quality of the studies. Data extraction and synthesis were carried out utilizing thematic content analysis.

**Results:**

The search identified 20 peer-reviewed studies meeting the inclusion criteria. Key themes were mapped into five distinct groups: perceptions, facilitators and barriers influencing the management of childhood obesity, as well as facilitators and barriers to enrolment into a weight management program. Parents often perceived obesity as a temporary condition, genetically determined and believed it should not be considered as a major health concern. Identified facilitators included the restriction of screen time, school involvement, goal setting, and enhanced child-parent communication. Conversely, barriers included lack of child motivation, peer influence, easy access to junk food, as well as parental denial, insufficient knowledge or control and logistical challenges.

**Conclusion:**

To tackle childhood obesity, it is essential to adopt a comprehensive strategy that fosters a supportive family environment. Successful initiatives should encompass nutritional education for both parents and children, increase access to healthy food choices, implement home-based programs, and improve the infrastructure that encourages physical activity. Additionally, cultural factors and technological advancements should be considered when designing these interventions.

**Systematic Review Registration Number:**

PROSPERO (CRD42024514219).

## Introduction

Childhood obesity is a growing public health concern cited as a universal epidemic by the World Health Organization (WHO).^1^,^2^ In 2022, it is estimated that 37 million children under the age of five were classified as overweight. Additionally, over 390 million children and adolescents aged 5 to 19 were reported to be overweight in the same year.^2^ The prevalence of overweight (including obesity) among this age group has experienced a significant increase, rising from 8% in 1990 to 20% in 2022.^2^ The prevalence of obesity among children, along with its associated health complications, contributes substantially to economic burdens, familial challenges, societal impacts, diminished academic performance, reduced health-related quality of life, and decreased life expectancy.^3–5^

Numerous interrelated factors were linked to childhood obesity including genetic predisposition, behavioral factors such as sedentary lifestyle and excessive food calories as well as socioeconomic and environmental influences such as insufficient places for physical activity or limited food choices.^6^,^7^ Industrialization, urbanization, and technological advancement have fostered an obesogenic environment characterized by the easy availability and affordability of low-cost, energy-dense, and nutrient-deficient foods or beverages, while opportunities for physical activity are often not integrated into daily routines.^5^,^8^

Childhood obesity increases the risk of non-communicable diseases such as hypertension, diabetes mellitus, musculoskeletal conditions, respiratory complications, as well as psychosocial challenges such as eating disorders, anxiety, depression, low self-esteem, and social isolation.^9^,^10^ Research indicates that children living with obesity are nearly twice as likely to experience multiple related comorbidities^11^ and have a fivefold increased risk of remaining obese into adulthood compared to their normal-weight peers.^12^ This trend contributes to a heightened burden of cardiometabolic diseases and an increased risk of premature mortality.^13^

Childhood obesity presents significant clinical and psychological challenges, yet global initiatives have not effectively curbed this growing epidemic.^14^ There is an increasing agreement among experts that interventions conducted at home, particularly those involving parental engagement in management strategies, can play a crucial role in helping children achieve a healthy weight.^15^,^16^ The dietary habits, food choices, and overall lifestyle of children are heavily influenced by their parents’ nutritional knowledge, eating behaviors, cultural and social factors, motivation for physical activity, and regulation of screen time.^17–19^ Previous studies have shown that parental understanding of childhood obesity management is multifaceted and varies throughout different stages of life.^20^ Families and healthcare professionals continue to encounter substantial obstacles in their efforts to combat childhood obesity.^21^

Numerous interventions aimed at addressing childhood obesity exist; however, only a limited number have shown significant effectiveness or sustained benefits. In a systematic review and meta-analysis conducted by Ho TJH et al, it was determined that school-based interventions could effectively lower body mass index (BMI) when they incorporated multiple components, such as physical activity, nutrition education, and parental involvement.^22^ Additionally, prior research indicated that healthcare professionals often encounter challenges when discussing childhood obesity with parents, stemming from various perceptions and beliefs, which can lead to delays in treatment.^23^ Chai LK et al found in their review that family-centered interventions, whether directed at parents alone or involving their children, were more successful in managing the weight of children living with overweight or obesity. These interventions typically included educational sessions focused on nutrition and physical activity, the development of positive parenting skills, role modeling, and strategies for managing challenging behaviors.^24^

Carroll C et al in their ethnographic review revealed that the involvement of children and their caregivers in obesity management services is influenced by their perceptions of the program’s content, acceptability, anticipated benefits, demands, communication strategies, goal setting, procedural steps, and monitoring metrics. The likelihood of completing an obesity management program increased for caregivers and children who recognized success in terms of behavioral changes, enhanced self-esteem, motivation, timely support, and engagement from peers, family, and school. However, barriers such as social stigma, feelings of guilt associated with failure, intervention costs, necessary dietary adjustments or travel, and a lack of role modeling from caregivers were identified.^25^

Given the complexity of childhood obesity management and the fundamental role of the family within the home, school or healthcare settings, understanding parental insights, enabling and limiting factors is essential to provide the best support and recommendations to this important public health problem. Therefore, the aim of this systematic review was to identify and synthesize the qualitative research literature regarding parental and caregiver perceptions, motivators, and barriers to the management of childhood obesity. In addition, it aims to explore their perceptions about referral, enrollment, or retention into a weight management program.

## Methods

### Protocol and Registration

This systematic review was prepared in line with the Preferred Reporting Items for Systematic Review and Meta-Analyses (PRISMA) guidelines.^26^ The review protocol was registered in the International Prospective Register of Systematic Reviews (PROSPERO) system (CRD42024514219).

### Eligibility Criteria

Any qualitative article aimed at exploring the perceptions, motivators, and barriers faced by parents and caregivers in managing childhood overweight and obesity was considered eligible for inclusion. Research articles were included if they satisfied the inclusion criteria outlined in Table 1.

**Table 1:** Inclusion Criteria of Selected Research Articles

Inclusion Criteria	
Study design	Qualitative studies or mixed quantitative ‎and qualitative components exploring the perception, motivators, and/or barriers for the management of childhood overweight & obesity OR enrollment or completion of a weight management program.
Population of interest	Parents and caregivers of children (≤ 18 years), any gender, overweight or obese.
Intervention or exposure	No intervention necessary.
Outcomes of interest	• Parental perceptions, facilitators, and barriers to the management of childhood obesity. • Parental facilitators and barriers to their children’s enrollment or completion of a weight management program.
Setting	No specific setting
Search period	2006–2023

The search parameters encompassed full-text publications in academic journals published between 2006 and 2023. There were no restrictions based on language or country of origin. The initial search resulted in 22,580 articles, which were subsequently imported into the EndNote bibliographic reference database. A total of 13,497 duplicates were eliminated through both manual and electronic methods.

### Data Source and Search Strategy

A broad literature search was undertaken employing a range of electronic databases, namely SCOPUS, PubMed, and Google Scholar covering the years 2006 to 2023. Search terms used are outlined in Table 2.

**Table 2:** Search Terms

OR	AND OR	AND OR	AND OR	AND OR	AND OR
Parent	Perception	Children	Overweight	Management	Systematic
Caregiver	Motivators	Adolescent	Obesity	Weight loss	Review
Mother	Barriers	Child	BMI	Program	Qualitative
Father	Experience	Childhood		Intervention	Thematic analysis
Family	Belief				Focus group
	Knowledge				Interview
	Opinion				In depth

### Data Extraction

Based on the inclusion criteria, an initial screening was undertaken to assess the relevance of studies. Titles and abstracts, and where necessary, full text, were screened independently by two reviewers (SM, AA) to identify papers of potential relevance. Any disagreements were discussed with a third author (VK) until a consensus was reached. Information was extracted based on specific study characteristics, including the author, year of publication, research aim, qualitative methodology, participant details, sample size, and study setting.

### Quality Assessment Method

The methodological quality of the studies was assessed utilizing the Critical Appraisal Skill Program (CASP) checklist.^27^ The tool consists of ten questions that address three main aspects: validity, confidence in the results, and the potential value of the study. Each question was rated as “Yes”, “Can’t tell”, or “No”. Two reviewers independently assessed each study using the checklist, with any disagreements settled through discussion. The quality scoring system was as follows: a score of 75% or higher is deemed high, 50–74% is classified as medium, and below 50% is regarded as low. No studies were excluded based on the quality assessment.

### Data Synthesis

The data generated through the systematic review above was produced using the thematic synthesis described by Thomas and Harden,^28^ which integrates the perspectives of parents and caregivers to establish key themes. Common descriptive themes were identified and coded with the assistance of NVivo12. Direct quotations from participants in the primary studies, along with the principal author’s interpretations of the review, were compiled based on their similarities in meaning. In instances where textual aggregation was not feasible, the results were conveyed in a narrative format.

## Results

### Study Selection

**Figure 1:**
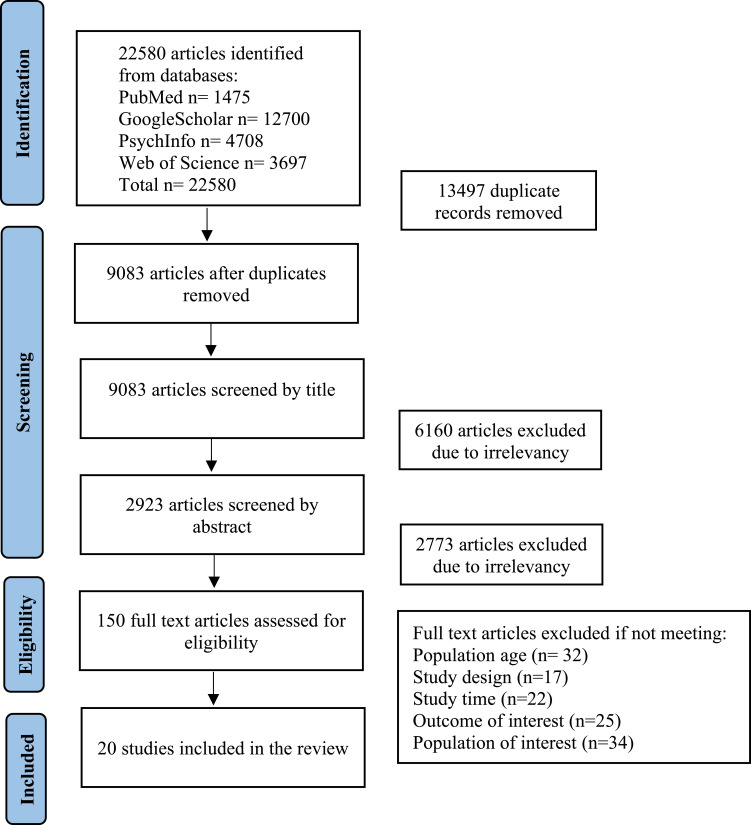
PRISMA diagram of the articles identified in each phase of review.

**Notes**: PRISMA figure adapted from Page M, McKenzie J, Bossuyt P et al. The PRISMA 2020 statement: an updated guideline for reporting systematic reviews. *Syst Rev*. 2021;10(1):1–11. Creative Commons.

Our search strategy yielded a total of 22,580 articles. Initially, the articles were screened based on their titles, resulting in the exclusion of 6160 articles. Subsequently, an abstract review led to the removal of an additional 2773 articles. A thorough examination of the full texts was conducted to evaluate eligibility criteria. Ultimately, 20 peer-reviewed journal articles satisfied the inclusion requirements Figure 1.

### Study Quality and Risk of Bias Assessment

The studies reviewed generally adhered to high quality standards, as evidenced by CASP scores between 80 and 100%, which suggest a low risk of bias. Nevertheless, a significant portion of the studies (70%) lacked a clear delineation and explanation of the relationship between researchers and participants, a factor that could have affected participants’ responses and introduced potential bias. Additionally, two studies did not employ suitable qualitative methodologies, specifically an online parenting discussion forum and an online survey with open text comments, thereby restricting a comprehensive examination of parental viewpoints regarding childhood obesity Table 3.

**Table 3:** Quality Assessment Using CASP Tool

	Borges et al (2018)^29^	Moore et al (2013)^30^	Visram et al (2013)^31^	Razi et al (2021)^21^	Brouwer et al (2017)^18^	Schmid et al (2020)^32^	Kinlin et al (2022)^16^	Owen et al (2009)^33^	Zuarub et al (2022)^34^	Alghamdi et at (2016)^35^	Kelleher et al (2016)^36^	Lindelof et al (2006)^37^	Douglas et al (2014)^38^	Sosa et al (2008)^39^	Alexander et al (2013)^40^	Rodríguez-Ventura (2014)^41^	Wild et al (2017)^42^	Merritt et al (2015)^43^	Schalkwijk et al (2010)^20^	Wild et al (2018)^44^
A clear statement of the aim	Y	Y	Y	Y	Y	Y	Y	Y	Y	Y	Y	Y	Y	Y	Y	Y	Y	Y	Y	Y
Appropriate Qualitative methodology	Y	Y	Y	Y	N	Y	Y	Y	Y	Y	Y	Y	Y	Y	Y	Y	N	Y	Y	Y
Appropriate research design addresses the aims	Y	Y	Y	Y	Y	Y	Y	Y	Y	Y	Y	Y	Y	Y	Y	Y	Y	Y	Y	Y
Appropriate recruitment strategy for the aims	Y	Y	Y	Y	Y	Y	Y	Y	Y	Y	Y	Y	Y	Y	Y	Y	Y	Y	Y	Y
Data collected in a way that addressed the research issue	Y	Y	Y	Y	Y	Y	Y	Y	Y	Y	Y	Y	Y	Y	Y	Y	Y	Y	Y	Y
Relationship between researcher & participants adequately considered	N	Y	N	N	N	N	N	N	Y	Y	N	Y	N	N	Y	N	N	N	Y	N
Ethical issues been taken into consideration	Y	Y	Y	Y	Y	Y	Y	Y	Y	Y	Y	Y	Y	Y	Y	Y	Y	Y	Y	Y
Data analysis was sufficiently rigorous	Y	Y	Y	Y	Y	Y	Y	Y	Y	Y	Y	Y	Y	Y	Y	Y	Y	Y	Y	Y
There is a clear statement of finding	Y	Y	Y	Y	Y	Y	Y	Y	Y	Y	Y	Y	Y	Y	Y	Y	Y	Y	Y	Y
The research is valuable	Y	Y	Y	Y	Y	Y	Y	Y	Y	Y	Y	Y	Y	Y	Y	Y	Y	Y	Y	Y
Quality score (%)	90	100	100	90	80	90	90	90	100	100	90	100	90	90	100	90	80	90	100	90

**Note**: Quality rating: <50% “low”, 50–74% “medium”, ≥75%.

**Abbreviations**: Y, present; N, not present.

### Characteristics of the Included Studies

Table 4 presents a summary of the characteristics of the studies included in this review. Each study incorporated either qualitative methods or a combination of quantitative and qualitative approaches. In total, data from 634 parents and caregivers were analyzed. Six studies were conducted in the United Kingdom, four in the United States, two in Canada, two in New Zealand, and one each in Australia, Georgia, Mexico, Denmark, and the United Arab Emirates. Fourteen studies employed interviews, while four utilized focus groups to gather insights on parental perceptions regarding childhood obesity. Additionally, one study leveraged online parenting discussion forums, and another collected data through open text comments via online platforms, telephone, or postal surveys. The majority of the children represented by the participating parents and caregivers are those who are living with overweight or obesity, with ages ranging from 1 to 18 years. Thirteen studies took place in community settings, including homes, while five were conducted in hospital environments and three in schools. Eight studies specifically examined the perceptions of parents and caregivers whose children had been referred to or had completed a weight management program.

**Table 4:** Summary Characteristics of Included Studies

	Authors (year)	Aim	Qualitative Method	Participant, Sample Size	Setting/location
1.	Borges et al (2017)^29^	Analyzed the child’s current health, child feeding routine, family knowledge of childhood obesity, family attitudes that influenced reduction or increase child’s weight.	1:1 Interview (n=13)	Main caregiver of obese child aged (6–10) years old.	Community (Southern region of Brazil)
2.	Moore et al (2013)*^30^	Explore perceptions, motivators, and barriers to successful completion of their children of ‘The Youngest loser’ weight management program.	Interview (n=10)	Parents of overweight/obese children (10–13) years (who completed The Youngest Loser weight management program	Community (Mississippi, USA)
3.	Visram et al (2013)*^31^	Examine the experiences of parents/guardians in weight management intervention ‘Balance It’	Interview (n=17)	Parents/guardians of overweight/obese children (4–17 years) who completed ‘Balance It’ program.	Home, community (Northern England)
4.	Razi et al (2021)^21^	Explore challenges experienced by parents in the care & management of childhood obesity.	Interview (n=18)	Parents of obese children aged (6–12) years.	Home, schools, community (Mashhad, Iran)
5.	Brouwer et al (2017)^18^	Explore parental challenges to managing childhood weight.	Online parenting discussion forums (n=206)	Parents/caregivers of children	Online parenting discussion forum (Australia, UK & USA)
6.	Schmied et al (2018)*^32^	Explore factors affecting parent engagement in a family-based childhood obesity prevention & control program.	Interview (n=22)	Parents/caregivers of children aged (2–11) years, BMI percentile >5% who were enrolled at ‘Family Wellness Program’	Community Imperial County (California)
7.	Kinlin et al (2022)*^16^	Assess feasibility, acceptability and factors affecting implementation of STOMP-EY obesity management intervention.	Focus groups.	Parents of obese (BMI≥97^th^ percentile) children aged (1–6) years old enrolled at STOMP-EY intervention.	Hospital Obesity management clinic (Toronto, Canada)
8.	Owen et al (2009)^33^	Explore parental perception of motivators & barriers to childhood weight loss.	Interview (n=21)	Parents of children attending the childhood obesity clinic.	Hospital Childhood obesity clinic (Bristol, England)
9.	Zuarub et al (2022)^34^	Explore views of parents on factors that might contributed to childhood obesity & perception of strategies to overcome barriers	In depth interviews (n=26)	Parents of 9–13-year-old children with overweight/obesity (BMI >85^th^ percentile) who attended government schools.	Schools (UAE, Sharjah, Dubai)
10.	Alghamdi et al (2017)*^35^	Explore motivations & barriers of parents participating in an outpatient, family-based, program to manage childhood obesity.	Interview (n=3)	Parent of children aged (2–18) years with overweight/obese enrolled at outpatient family-based program.	Public; library (Ontario, Canada)
11.	Kelleher et al (2019)*^36^	Explore families’ barriers & facilitators for their children referral/completion of a community weight management program (W82GO-community)	Interviews (n=9)	Families of overweight/obese children (BMI ≥98^th^ centile) aged (5–7) years.	Community; healthcare offices, local sports, or community center (South & West of Ireland).
12.	Lindelof et al (2010)^37^	Explore facilitators and barriers to lose weight among adolescents with obesity.	Field observations & interviews (n=15)	Parents of obese adolescents aged 13–16 years who participated in weight management summer camp.	Community; homes (Denmark)
13.	Douglas et al (2014)^38^	Explore parental perception of the determinants of early childhood obesity and beneficial management strategies.	Focus groups (n=34)	Parents & carers of children aged 3–4 years old	Community (North-East Scotland)
14.	Sosa et al (2015)^39^	Explore maternal perception in terms of causes, consequences, strategies to manage childhood obesity	Focus groups (n=23)	Mexican American mothers of a child aged 5–12 years	Community (Bryan, Texas, USA)
15.	Alexander et al (2014)^40^	Explore parental perceptions of childhood obesity risk factors, health concerns, barriers & facilitator of management.	Interview (n=12)	African American parent of children aged 8–11 years attending the targeted elementary school.	Community; home, school (East-Central, Georgia)
16.	Rodríguez-Ventura et al (2014)^41^	Identify weight perception, barriers to lose weight, beliefs, habits, and strategies for weight management.	Focus groups (n=22)	Parent of overweight/obese (BMI ≥ 85^th^ percentile) children aged (10–18) years	Hospital; pediatric endocrinology unit (Mexico)
17.	Wild et al (2020)*^42^	Identify barriers & facilitators to engagement in weight management program.	Open-text comments (n=71, completed the survey)	Parents of children with obesity >11 years old.	Online, telephone, post (Taranaki, New Zealand)
18.	Merritt et al (2019)^43^	Explore caregivers’ perceptions, attitudes, and behaviors to children’s diet, exercise, and weight.	Interview (n= 39)	Caregivers of children aged 2–11 years with overweight/obesity through local children’s centers and market research company.	Community; home, telephone (East Sussex, UK)
19.	Schalkwijk et al (2015)^20^	Identify barriers to making lifestyle changes and experiences in lifestyle interventions.	Interview (n=24)	Parents of overweight/obese children aged 4–12 years	Youth health care, Primary health care center, pediatrics (Netherlands)
20.	Wild et al (2020)*^44^	Explore barriers to attendance/retention in weight management program	Interview (n=64)	Parents/caregivers of children aged 4–16 years with overweight/obesity (BMI≥91^st^ centile)	Hospital, Community; home, workplace (Urban-rural, New Zealand)

**Notes**: *Studies explored the perceptions of parents and caregivers whose children have been invited, referred to or completed a weight management program.

**Abbreviations**: BMI, Body Mass Index; STOMP-EY, the SickKids Team Obesity Management Early Years.

### Thematic Findings

The thematic analysis facilitated the organization of study responses into three primary themes, each encompassing additional subthemes: knowledge and beliefs, motivators, and barriers identified by parents and caregivers in the management of childhood obesity. The subsequent phase of analysis concentrated on the facilitators and obstacles that parents encountered when enrolling their children in weight management programs Table 5.

**Table 5:** Summary of Study Themes Related to Parental Perception, Facilitators, Barriers to the Management of Childhood Obesity

**Theme**	**Description**	
	**Management of childhood obesity**	
**Perception**	Childhood obesity is genetically determined. Medical conditions cause obesity. Obesity is not a health concern. Thinness is perceived as illness. Food restriction affects children’s growth. Overweight or obesity is a transient state. Healthy food is already served at home.	
**Facilitators**	Parents. positive attitude/support/communication. Involvement of exercise specialists. Motivation to engage in physical activity. Healthy eating behaviors & defined portions/substitutions. Active school involvement. Good parenting skills & food behavior. Monitor children’s food intake. Advise from nutritionist.	
**Barriers**	**Child factors** Child stubbornness. Child secretiveness “sneak eating”. Purchasing junk food. Low nutritional knowledge. Peer pressure. Picky eaters. Disliking fruits & vegetables. Lack of awareness of self-determination needs. Child’s intrinsic motivation. Snacks eating. Lazy & not liking exercise. Stressful bond with parents. Watching TV/videogames. COVID-19 impact on PA. **Other factors**: Unsupportive nannies. Limited nutritional education in school. Conflicting messages from family/friends/school/. Culture impact. Cost of food. Marketing of unhealthy food. Low socioeconomic. Advancement of technology reduced PA.	**Family factors** Non-acceptance of term “obese”. Parental denial. Fear of diagnosis. Parent conflict in management. Interference of other family members. Limited parental engagement due to busy jobs. Parents not being the ideal role model. Lack of knowledge/skills to manage lifestyle change. Lack of knowledge on BMI/growth chart. Blaming children. Lack of knowledge on healthy cooking/diet. Lack of screening/motivation of caregivers. Insufficient opportunities given to children for PA. Lack of control over sedentary behavior (computer/TV). Allowing bad foods/treats Convenience of foods due to busy day and limited time Serving an unhealthy breakfast. Skipping/replacing main meals. Fear of emotional or eating disorders. Unable to control the outside food that the child brings. Lack of knowledge on age-appropriate portion size. Lack of parental supervision. Buying fast food to compensate guilty feelings. Lack of knowledge on correct amount of exercise. Restrict PA due to safety or weather. Feeling guilt with diet restriction. Lack of parental psychological support. Using food as reinforcement of behavior. Lack of knowledge on consequences of obesity (physical/social/emotional).
	**Enrollment/completion of weight management program**	
**Facilitators**	Improving parental knowledge & skills to guide children in eating choices. To gain self-confidence & overcome challenges. Working with groups & peers. Supportive staff. Working as a whole family. Perceived benefit, flexibility, affordability & accessibility of the program. Family support for children to participate. Perception of direct/indirect benefits of the program. The need for professional weight management support. Open/relaxed/non-judgmental communication. Exchange experiences with other families. Hands-on activities Focus on lifestyle choices rather than weight loss. Visual & practical group sessions. Need for support & outside help. Desire to improve child’s health. Defined goal & incentives. Able to get personalized counselling.	
**Barriers**	Conflict with schedule/transport. Unclear or sufficient information. Limited fit between intervention & needs. Limited value of group sessions. No psychological support for families. Stigmatization for referral to obesity program. Cost or location of program. Parents prefer home-based programs. Difficulty putting program advice into practice. Not suitable for age, non-relevant, not a priority. Adverse life/family stressors. Believes that young children do not need weight management programs. Fear children develop self-esteem issues. Previous negative experience.	

**Abbreviations**: PA, Physical Activity; BMI, Body Mass Index.

### Perceptions and Beliefs

Parents and caregivers frequently view a child’s weight or size as predominantly influenced by genetic factors, which leads them to believe they have little responsibility in addressing the child’s risk for weight-related problems. Additionally, they have expressed the opinion that an increase in weight, provided there are no physical or psychological issues, should not be considered a major health concern.^18^,^29^,^39^,^44^

Thinness is often regarded as a health issue, prompting many parents and caregivers to assert that food should not be limited, as it is essential for a child’s development.^41^,^43^ Parents held the belief that obesity is a temporary condition and that children will eventually outgrow it.^18^,^44^ Despite the significant variability in how parents and caregivers characterize their children as obese, this characterization is not typically associated with BMI or clinical indicators.^18^,^31^,^38^,^40^ Rather, obesity is often described in terms of physical appearance, using descriptors such as “stocky”, “broad”, “chubby”, or “chunky”.^18^,^29^,^31^,^38^,^39^,^41^,^44^ It is often not recognized until later stages when complications arise, such as asthma or acanthosis nigricans.^18^,^34^,^35^ While some parents and caregivers acknowledge psychological effects like low self-esteem, bullying, and social isolation, only a few are able to recognize the potential long-term physical consequences.^35^,^40^

I would not worry about it. She is two years old, and I personally think watching a VERY young child’s weight is absolutely ridiculous (unless he or she is wayyy overweight or it’s obviously affecting their health).^18^

having visited for something else entirely different and then being told kind of ‘your child’s obese and we are going to refer you and just doing it front of him […] it was just even in the way that it was delivered, and I was kind of not expecting it. I mean, I can see that he’s, he’s a bit chunky, but I just, I do not know […] [the referral] was a bit off-putting.^42^

### Motivators and Facilitators

Parental engagement, along with their attitudes and support, has been identified as a crucial factor in the management of children’s weight.^34^,^39^,^41^ Parents and caregivers emphasized that promoting healthy eating habits, as well as regulating and defining portion sizes for their children, are essential components for fostering nutritious eating practices.^29^,^34^,^39^,^40^ Furthermore, some parents recognized the importance of managing screen time, particularly television viewing, as a means to reduce sedentary behavior and food consumption, thereby aiding in weight management.^39^,^41^ Active participation in school activities was also highlighted by parents and caregivers as an effective strategy for encouraging healthy eating and physical activity.^41^,^42^ Additionally, several parents noted that guidance from healthcare professionals, exercise specialists, or nutritionists in developing tailored healthy lifestyle programs serves as a significant facilitator in addressing childhood obesity.^34^

Having a nutritionist’s advice will contribute to educating my child about the importance of eating healthy and the way to build a balanced meal plan.^34^

The school administration can participate by publishing awareness campaigns in schools to reinforce the conviction of children about the importance of healthy meals for their physical health.^34^

### Barriers

Two subthemes were distinguished as common barriers perceived by parents and caregivers. The first pertains to factors related to the child, while the second concerns parental and family dynamics.

### Child Factors

Parents reported that children’s stubbornness and tendency to conceal junk food purchases hindered efforts to manage weight.^21^,^35^,^37^ Additionally, issues such as selective eating habits,^18^,^35^,^43^ aversion to fruits and vegetables,^18^,^41^,^43^ and frequent consumption of unhealthy snacks^34^,^35^ have posed significant challenges to the promotion of healthy lifestyle choices.

If they’ve got £1 in their pocket, I can’t stop them [from] buying a packet of crisps and a chocolate bar when they want it if the’ve got the money. I just try and say to them that there’s better things to [eat?].^38^

Parents and caregivers expressed worries regarding the child’s intrinsic motivation,^37^ noting tendencies towards laziness and a disinterest in physical exercise.^18^,^37^ Additionally, there were concerns about the child’s limited nutritional knowledge and a lack of awareness regarding self-determination needs.^35^,^41^ Some parents and caregivers recognized that extended periods of television viewing and video game play could promote sedentary lifestyles, thereby displacing time that could be allocated for physical activity.^34^,^37^,^43^ Furthermore, peer pressure was identified as a significant factor affecting the child’s food choices.^34^

As I noted earlier, his sudden obsession with fast food is the main factor affecting his weight. Perhaps this new eating habit was influenced by social media and his school fellows.^34^

### Family Factors

Non-acceptance of the term “obese”, parental denial or fear of diagnosis were identified as key barriers to children weight management.^29^,^31^,^36^,^40^,^41^,^43^,^44^ Parents expressed concerns about not being adequate role models^20^,^34^,^38–40^ and noted that conflicts among family members regarding management strategies further complicated efforts to control their children’s weight.^18^,^20^,^21^,^38^ Additionally, a lack of parental knowledge and skills related to lifestyle modifications,^35^,^38^ BMI and growth charts,^18^,^36^ and healthy cooking and dietary practices was seen as a barrier to successful weight management.^21^,^34^,^38^,^41^ Parents and caregivers acknowledged that their busy work schedules limited their ability to engage in healthy behaviors,^21^,^32^,^34^,^35^,^38^,^41^ leading to challenges such as insufficient opportunities for physical activity^21^,^34^,^38^,^43^ and the convenience of unhealthy food options.^38^ Furthermore, inadequate resources, including space, financial constraints, and access to playgrounds, were cited as additional barriers. Lastly, a lack of parental motivation to monitor or intervene in their children’s weight issues, coupled with a tendency to blame the children for their obesity, was also highlighted as a significant challenge.

Well, my child is very interested in fast food, of course, I do not give them to her, but my husband sometimes buys them, and I really cannot stop him.^21^

Since I am a working mother, I do not have much time to cook healthy meals daily. Most of our meals consist of frozen food or food filled with starchy foods such as rice and pasta, in addition to our heavy reliance on takeaway.^34^

Some time ago we were guests at my brother’s house, my child was overeating, and I asked him to stop. But then his uncle and aunt complained that why you are treating the child like that?!. what can we do in this situation…? You tell yourself they are older, and you should respect them.^21^

## Engagement or Retention in Weight Management Programs

### Facilitators

Parents were more inclined to enroll their children in a weight management program when they were motivated to enhance their children’s health^32^,^35^,^36^,^42^ and recognized the advantages of such programs, including the ability to better understand and guide their children’s dietary choices while also mitigating weight-related stigma.^16^,^32^,^35^,^42^,^44^ The aspect of convenience was crucial, encompassing not only the accessibility of the program but also its affordability and flexibility.^16^,^32^,^35^,^42^,^44^ Furthermore, parents believed that the competencies of the staff, characterized by qualities such as openness, relaxation, and a non-judgmental attitude, significantly influenced their decision to participate in or complete the program.^30^,^35^,^36^,^42^ Engagement was further bolstered by hands-on, practical, and visual family-oriented strategies, along with clearly defined goal setting.^30^,^35^,^36^ Additionally, some parents found motivation in developing skills through peer learning and sharing experiences with other families.^30^,^35^,^36^

I guess being around children that were like her and with [staff] and being so positive with them. I think they gave her confidence.^30^

[I enrolled] because my daughter, she was told her was pre-diabetic, that’s why, in fact that’s why they referred us our choice for her, because her insulin level was high.^32^

The biggest thing for me was the idea of the whole family. It was not just centering him out in any way. It was all of us. We’re all on board; we’re all learning the same things and the kids are learning it in a different way than we’re learning.^35^

I’d have loved to have come away with two or three sample meal plans or shopping lists or what to get in your Chinese takeaway. I think there could have been more practical help. Those practical sessions were brilliant. Sometimes we just listened to a session without being given actual examples and you could see some were getting bored towards the end.^36^

### Barriers

Various obstacles may have prevented parents from enrolling their children in a weight management program. For instance, busy or conflicting schedules,^30^,^32^,^35^,^42^ along with a lack of motivation^35^ or resources^31^,^36^,^44^ such as financial means or transportation, have been noted. Additionally, issues related to weight bias, stigma, or prior negative experiences have also been highlighted.^44^ Structural barriers associated with the program included unclear or inadequate information,^16^,^42^ a perceived unsuitability for younger participants,^42^ and the program being offered in a facility rather than in a home-based setting.^42^,^44^ A significant number of parents identified the absence of support from family or partners as a major hindrance.^30^,^32^,^35^

…definitely just logistics of getting her there…making it work with a very, very busy schedule, especially once school started.^30^

Kids are mean, and I didn’t want my daughter to have to think of bullying. I did not want her to have to go through life being self-conscious; I want her to grow happy. That’s what motivated me.^35^

I just thought it was a dietitian, but I didn’t know that it had more than just a dietitian, so there was a social worker, a pediatrician, there was physical education person, and all those things.^16^

## Discussion

This systematic review examined parental perceptions, facilitators, and barriers to the management of childhood obesity, as well as their views on the enrollment or retention in weight management programs. A total of twenty peer-reviewed articles were analyzed, encompassing 634 participants. The majority of the studies employed qualitative methods, including interviews and focus groups. Most of the research was carried out in community settings across different countries.

Our analysis, consistent with earlier research,^45^ identified a range of parental beliefs regarding the origins of obesity. These beliefs encompassed ideas of genetic determinism and the impact of medical conditions, while often overlooking the multifaceted nature of obesity and the role of lifestyle choices. Many parents struggled to articulate what constitutes overweight or obesity, tended to see obesity as a temporary condition, and sometimes regarded children with larger body frames as a sign of affluence.^46^

### Barriers to Childhood Obesity Management

Notable barriers to managing obesity were categorized into factors related to children and families. Parents identified issues such as children’s stubbornness, selective eating habits, insufficient nutritional knowledge, sedentary lifestyles, lack of motivation, and peer influence as child-related factors. Prior studies have indicated that sleep patterns, stress levels, and coping strategies may also significantly contribute to the onset of obesity in children.^47^ In terms of family-related factors, a lack of nutritional understanding and difficulties in interpreting BMI, along with the challenges faced by working parents in providing opportunities for physical activity, were noted. Additionally, previous research highlighted parental denial, fear of a potential diagnosis, and a lack of knowledge as significant barriers.^48^

Cultural factors, including beliefs and attitudes, are believed to significantly influence the lifestyle choices of children. In regions such as the Middle East, it is common for families to employ expatriate nannies from developing nations to care for their children. These nannies frequently lack adequate health literacy, which hinders their understanding of the importance of nutritious diets and the adverse effects of unhealthy eating habits and inactivity. Consequently, they may inadvertently shape children’s dietary practices through their own behaviors.^49^ Additionally, it is possible that cultural perspectives lead parents to not recognize childhood obesity as a health concern.^50^ Certain cultural traditions and preferences, particularly for calorie-dense foods and sedentary activities, have been linked to increased rates of childhood obesity among specific ethnic populations.^51^

### Facilitators to Childhood Obesity Management

Our analysis revealed multiple facilitators that present valuable opportunities for intervention strategies. These factors encompassed a positive parental attitude, supportive communication, and effective engagement; participation of exercise professionals; motivation to partake in physical activity, healthy dietary practices, and appropriate portion control or substitutions; active participation in school initiatives; proficient parenting skills and food-related behaviors; regulation or management of children’s food intake; and guidance from nutritionists. The collaboration of exercise professionals, nutritionists, and educational institutions, combined with the provision of information and support networks, significantly increases the chances of effective obesity management.^52^,^53^

Empowering children through their active involvement in goal setting and decision-making regarding health-related behaviors emerged as a key facilitator in our review. This aligns with earlier studies indicating that when children participate in decision-making, their motivation and commitment to healthy behaviors tend to improve.^54^ Enhancing this involvement can be achieved through education, peer support, and active participation in the planning and execution of interventions. Additionally, establishing clear rules and boundaries around food, along with modeling healthy eating, can significantly impact children’s food choices and eating habits.^55^

The current review identified the belief that “healthy food” is already served at home. This perception may stem from a lack of understanding regarding the nutritional value of the food consumed, as well as cultural traditions or taboos. Although some families may offer healthy food choices, numerous others may not possess the necessary knowledge or resources to provide such options.^56^

A key facilitator highlighted in this review is the positive attitude, support, and communication from parents. When parents exhibit a favorable outlook on healthy eating and maintain supportive dialogue with their children, it cultivates an environment conducive to healthy behaviors. This observation aligns with earlier studies that underscore the significance of parental engagement in interventions aimed at managing obesity.^57^

Additionally, our review identified active participation in schools as another key facilitator. Schools significantly influence children’s behaviors and attitudes regarding food and physical activity. Prior studies have shown that interventions implemented within schools, such as nutrition and physical education classes, along with the promotion of healthy food options, contribute to an environment that encourages healthy behaviors.^52^,^53^

Our review highlighted the critical role families play in managing childhood obesity. A home environment characterized by fewer “obesogenic” elements, which promotes healthier food choices and encourages self-regulation, proves to be more effective than strategies that focus exclusively on the child.^58^ An interventional study conducted by Golan et al^59^ revealed that, over a follow-up period of up to seven years, children whose parents participated in the intervention experienced greater weight loss and maintenance compared to those in a child-only group. Additionally, Berkowitz et al^60^ emphasized that family support and active involvement in decision-making significantly enhance children’s motivation, engagement, and adherence to weight management initiatives.

### Enrollment or Completion of a Weight Management Program

Our review revealed that a number of parents favor home-based programs. This inclination may stem from various factors, including convenience, privacy, and the capacity to incorporate the program into their everyday lives. Previous research has highlighted the effectiveness of home-based interventions, underscoring the necessity of providing a variety of intervention options to meet different preferences.^61^

Home-based programs offer numerous benefits compared to hospital-based interventions, particularly in addressing access barriers and enhancing outreach. For many families, hospital-based interventions may present challenges related to transportation, time, and financial limitations. In contrast, home-based programs can be tailored to meet the specific needs of each family, providing continuous support in a familiar and comfortable environment. This approach often results in higher participation rates and better adherence to intervention programs. Research has demonstrated the success of home-based interventions in improving health outcomes for children.^62^ Consequently, it is essential for healthcare professionals and policymakers to prioritize the implementation of home-based interventions to broaden the impact and effectiveness of initiatives aimed at combating childhood obesity.

Socioeconomic inequality presents considerable obstacles to accessing weight management programs. A systematic review revealed that families with lower incomes exhibited reduced participation and retention rates in weight management initiatives in the United States.^63^ Additionally, factors such as time limitations, resource availability, and commuting challenges were identified as significant barriers.^64^ Further studies have shown that the educational attainment of parents affects their ability to manage their children’s behavior and their motivation to maintain involvement in these programs.^65^

### Implications to Practice and Research

A comprehensive strategy is essential to tackle childhood obesity effectively. Healthcare professionals need to actively evaluate children’s lifestyles and behaviors while introducing interventions that focus on various determinants, environmental influences, and psychological aspects such as awareness and motivation. Additionally, the role of parents is vital; their supportive attitudes and effective communication can significantly encourage healthy habits in their children. Interventions aimed at modifying parental purchasing decisions and enhancing the availability of nutritious food at home have proven beneficial in improving children’s dietary practices.

Addressing challenges such as stigmatization, financial constraints, peer influence, and cultural beliefs is crucial. This necessitates the implementation of strategies aimed at improving parental understanding and self-assurance. Developing effective parenting skills that enable children to make autonomous decisions, fostering a supportive mealtime atmosphere, utilizing positive reinforcement, and serving as role models are all examples of effective interventions.

Collaboration among healthcare professionals, educational institutions, policymakers, and community stakeholders is essential for developing tailored interventions and innovative approaches that foster positive health outcomes. Furthermore, implementing policy changes—such as limiting the marketing of unhealthy foods and enhancing nutritional education—can help establish healthier environments, thereby supporting comprehensive efforts to address childhood obesity.

## Conclusion

This systematic review provides important insights that can guide the formulation of future interventions and policies aimed at decreasing childhood obesity rates. Cost-effective interventions involve engaging children in the decision-making process, establishing a robust family support system, and offering home-based programs while considering the adverse effects of culture and technology. Collaboration among families, healthcare professionals, and educational institutions is essential for achieving sustainable positive outcomes.

## Data Availability

All data generated or analyzed during this study are included in this manuscript. Further inquiries can be directed to the corresponding author.
